# High-resolution global population projections dataset developed with CMIP6 RCP and SSP scenarios for year 2010–2100

**DOI:** 10.1016/j.dib.2022.107804

**Published:** 2022-01-06

**Authors:** Niklas Boke Olén, Veiko Lehsten

**Affiliations:** a Centre for Environmental and Climate Research, Lund University, Sölvegatan 37, Lund SE-223 62, Sweden; b Swiss Federal Institute for Forest, Snow and Landscape Research WSL, Switzerland; c Department of Physical Geography and Ecosystem Science, Lund University, Sweden; d Department of Economics, Geography, Law and Tourism, Mittuniversity, Sweden

**Keywords:** Population, Scenarios, IPCC, Global, Modelling

## Abstract

We present a novel, global 30 arc seconds (∼1 km at the equator) population projection dataset covering each year from 2010 to 2100 that is consistent with both country level population and gridded urban fractions from the Coupled Model Intercomparison Project 6 (CMIP6). While IPCC population projections until 2100 are available at country level for Socio-Economic Pathways (SSPs), land cover (including the urban fraction) is only available for Representative Concentration Pathways (RCPs). To perform simulations of e.g., future supply and demand for agricultural products, fine scale projections of population density are needed for combinations of SSPs and RCPs. Therefore, we generated a 30 arc seconds dataset consistent with both SSPs and RCPs within the framework of the IPCC. This data set is useful in applications where spatially explicit projections of aspects of global change are investigated at a fine spatial scale. For example, if a link function between night-time lights and population density is found based on current satellite images and recent population density data, a projection of night-time light lights can be generated by using this link function with our projected population density. Such a projection can for example be used to evaluate the potential for future light pollution.

## Specifications Table

**Table:** 

Subject	Global and Planetary Change
Specific subject area	Population projection
Type of data	Gridded dataset
How data were acquired	Simulated
Data format	Annual files of population density at 1 km resolution in Geotif format.
Parameters for data collection	The input data were selected to contain the necessary data to estimate the future population in relation to IPCC CMIP6 scenarios.
Description of data collection	Input data to model future population
Data source location	SSP population - https://tntcat.iiasa.ac.at/SspDb/ RCP urban fraction - https://luh.umd.edu/ WorldPop – Unconstrained global mosaics - https://www.worldpop.org/geodata/summary?id=24767 Roads - https://sedac.ciesin.columbia.edu/data/set/groads-global-roads-open-access-v1 Water bodies - https://sedac.ciesin.columbia.edu/data/set/grump-v1-national-admin-boundaries/data-download (created with national boundaries data) Country borders – http://thematicmapping.org/ Urban extent - https://www.researchgate.net/publication/339873537_MGUP_annual_global_2001_2018.
Data accessibility [1]	Repository name: DataGURU Data identification number: https://doi.org/10.18161/global_popcount.201610 Direct URL to data: https://dataguru.lu.se/app#worldpop
Code accessibility [2]	Repository name: GitHub Direct URL to code: https://github.com/niklasbokeolen/world_population

## Value of the Data

• This dataset allows to generate projections (*i.e.* estimations of future development) of any issue related with global change which requires population density as a parameter.
• Researchers working in global change issues but also spatial planners at state levels are the intended end-users/stakeholders of this dataset.
• This data can be used as an input data set for studies evaluating aspects related to global change. For example, air pollution depends on the social settings in the development scenario, such as per capita income or mechanisation level, but at a fine spatial scale it also depends on population density, which has not been available before.

## 1 Data Description

### 1.1 Dataset

We generated population projections [1] for 6 combinations of SSPs and RCPs, see below. All of them can be accessed at the DataGURU server (https://dataguru.lu.se/, short link to this data set: https://dataguru.lu.se/app#worldpop) which also allows basic pre-processing such as selection of certain years, temporal averaging and spatial cropping. After selecting an SSP/RCP combination, the data can either be pre-processed by the DataGURU server or downloaded directly via ftp. The data are stored as one geotiff (.tif) file for each scenario combination and year with the datatype FLT4S and NA-value as -9999. They are in a longitude latitude projection with WGS84 as the datum. The files are named to correspond to year, SSP and RCP scenario numbers (information in brackets are replaced by year or number).

“CMPI6wp_grid_pop_count[year]_SSP[SSPnr]_ RCP[RCPnr]”.

Data ranges from 2010 until 2100 and are available for the six different SSP RCP combinations (SSP1-RCP2.6, SSP2-RCP3.4, SSP3-RCP7.0, SSP4-RCP3.4, SSP4-RCP6.0, SSP5-RCP8.5).

The naming of the datafiles will differ if advanced features of DataGURU such as cropping are used. The code used to generate the dataset is available on GitHub [2].

**Fig. 1:**
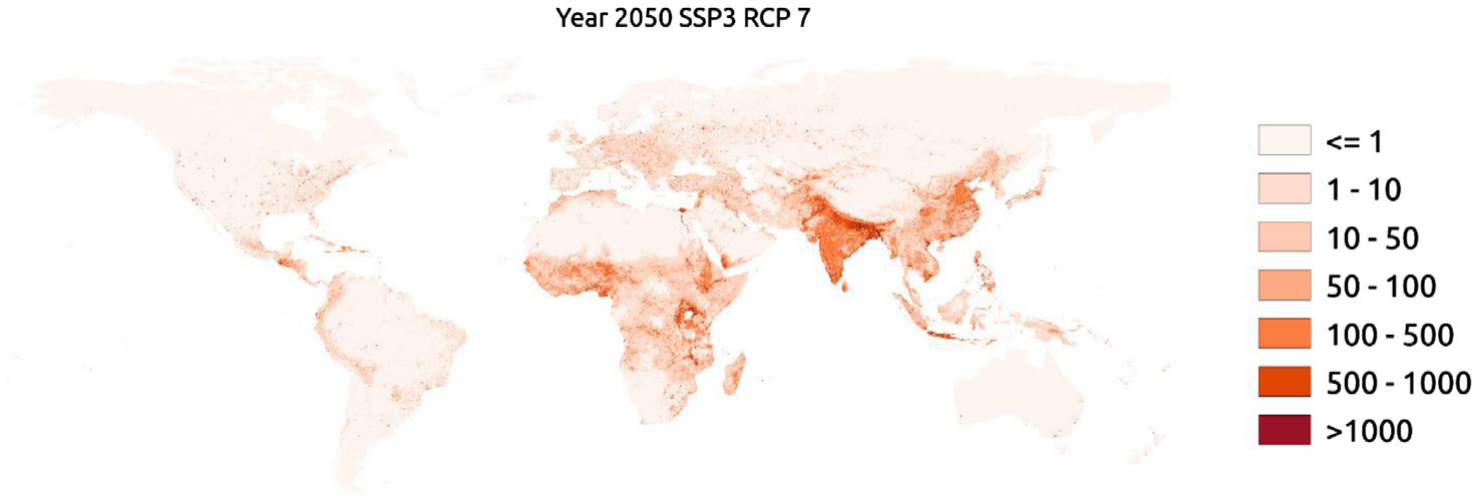
Flow chart describing the method used to distribute population. Example made with artificial numbers. The smaller grid cells correspond to 30 arc second pixels and the full grid (9x) represents one 0.25-degree pixel, we also (for the sake of the example) assume that the full grid corresponds to one country. Green boxes with rounded corners indicate input data. The upper part represents the year 2010 and the lower one represents the procedure performed for the year 2011. For the year 2012 this process is repeated with the 2012 input data and the 2011 result data.

Fig. 1 is a conceptual figure showing the steps taken to create the resulting dataset. It is a flow chart describing the method used to distribute population. Example made with artificial numbers. The smaller grid cells correspond to 30 arc second pixels and the full grid (9x) represents one 0.25-degree pixel. We also (for the sake of the example) assume that the full grid corresponds to one country. Green boxes with rounded corners indicate input data. The upper part represents the year 2010 and the lower one represents the procedure performed for the year 2011.

**Fig. 2:**
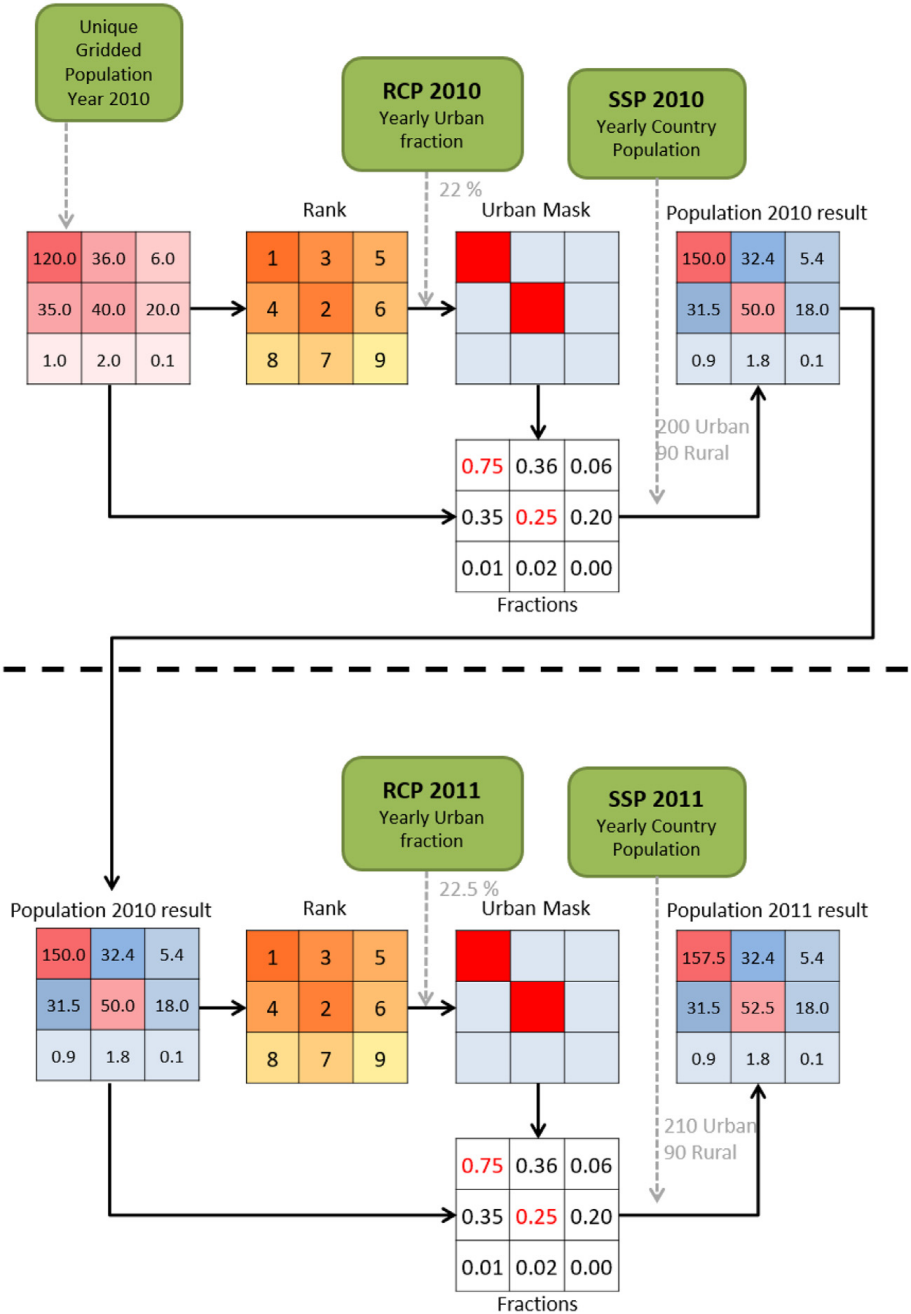
An example map of the population grid for year 2050 for SSP 3 and RCP 7. For visibility purposes the colour range has been restricted to be between zero and 1000, meaning that every pixel with 1000 or more people will be shown in the same way (dark red).

An example map of the population grid for year 2050 for SSP 3 and RCP 7. For visibility purposes the colour range has been restricted to be between zero and 1000, meaning that every pixel with 1000 or more people will be shown in the same way (dark red). Fig. 2 has been made with the resulting dataset found at DataGURU.

**Table 1:** Input datasets used to grid future populations.

Name	Spatial domain	Temporal domain	Type	Source
SSP population scenarios	Country	2000-2100	Continuous	SSP Database [7]
RCP Urban fraction	0.25 degree	2010-2100	Raster	LUH2-v2f (luh.umd.edu) [9]
WorldPop	30 arc-second	2010	Raster	https://www.worldpop.org/geodata/summary?id=24767ww.worldpop.org[6]
Roads	Global	1980-2010	Polylines	gROADSv1 [10]
Water bodies mask	Global	2000	Polygons	GRUMPv1:National-Administrative-Boundaries [12]
Country Borders	Global	2008	Polygons	Sandvik [13]
Urban Extent	Global	2001	Raster	MGUP [11]https://www.researchgate.net/publication/339873537_MGUP_annual_global_2001_2018

List of the input raw datasets used to create the population projections.

### 1.2 Raw data

The complete information on the raw data can be found in Table 1.

## 2 Experimental Design, Materials and Methods

Here, we present a yearly gridded population projection for 2010–2100 for the globe consistent with both the SSPs population and the RCPs urban fractions [3] based on the data for the Coupled Model Intercomparison Project Phase 6 (CMIP6) experimental design. The CMIP framework is one of the fundamental elements for climate change studies into the future. It combines and coordinates the design of global climate model simulations [4]. Our work is based and updates on the African future population dataset for CMIP5 projections developed by Boke-Olén et al. [5]. The population dataset, presented in this paper, is useful for global scale studies using gridded RCP land use and climate data in relation to gridded population estimates, for example to assess future supply and demand of food.

The method is based on distributing country level Shared Socioeconomic Pathway (SSP) urban and rural population projections onto a 30 arc seconds (∼1 km at the equator) grid. The method is designed to assure that the created gridded population is also conforming to the Coupled Model Intercomparison Project Phase 6 (CMIP6) Representative Concentration Pathways (RCP) urban fraction grid at 0.25°. We used global population data from the WorldPop Project [6] for year 2010 as a starting point for the population projections. The starting dataset was combined with population centre of gravity (one way to describe population centres using both spatial distribution and density [6]), distance to road, and distance to urban areas to allow each pixel to be ranked uniquely into urban or non-urban (see section below). A list of the used datasets can be found in Table 1.

The future population distribution was modelled to be consistent with both the CMIP6 RCP-specific urban fraction dataset (LUH2-v2f, luh.umd.edu) [9] and the country level SSP population and urban fraction scenarios from the SSP database [8]. The RCP urban fractions (LUH2-v2f) come as six different scenario combinations which are all used within this study to create separate population projections. The scenario combinations are SSP1-RCP2.6, SSP2-RCP3.4, SSP3-RCP7.0, SSP4-RCP3.4, SSP4-RCP6.0, SSP5-RCP8.5.

The WorldPop gridded population datasets for year 2010 were used as a starting point for the population projections. The water body mask polygon dataset was converted to raster with an output resolution of 30 arc-seconds. This mask was subsequently used to remove water bodies from the population dataset. While this step was not necessary as the WorldPop dataset already contains a sufficiently fine scaled water body mask, we report here that we performed this step to allow replicating our work. While this step (where we use an older water bodies mask) might influence the projected population on single pixels (in case the two datasets disagree on the extend of water), we expect this not to have a measurable effect. Moreover, the number of people within an administrative unit is not affected. To make sure that each 30 arc-second pixel of the initial dataset was unique within each 0.25-degree pixel we added the information of distance to road, distance to urban extent and distance to centre of gravity to the initial dataset. The three added datasets were each rescaled and inversed to be between 1.0·10^−5^ and 1.1·10^−5^ were values closer to the road, urban extent, or centre of gravity had a higher value, similar to Boke-Olén et al.[5]. The created dataset of semi-unique values per 0.25-degree pixel is from now on referred to as unique population dataset.

### 2.1 Population distribution

To distribute the urban and rural population separately we need to separate the two classes. This is done by using the unique population datasets as the starting point together with the urban fraction (Fig. 1). We rank each 30 arc-seconds pixel within each urban fraction grid cell and use this information to decide which are urban. The number of urban pixels within each 0.25-degree cell is calculated based on the urban fraction and is rounded to the nearest integer. The created urban pixels are used per country to distribute the urban and rural population values assuming the same relationship as in the unique population dataset. Using this method, we assure that the population size order between pixels remains if they are in the same country and both either urban or rural. This is illustrated in the example in Fig. 1 where the two urban pixels still have the same ratio of population sizes (3:1) relative to each other after the population allotment. Once the population has been distributed for the first year, the new created dataset will be used as input for the next year thus creating a loop (Fig. 1). An example of the global population dataset for SSP 3 and RCP 7 for year 2050 is displayed in Fig. 2.

### 2.2 Uncertainty

The population projections were created using a deterministic method hence the uncertainty of the output is solely caused by uncertainty in the input data (Table 1). Our method of creating a unique starting point by adding rescaled distance to road and urban extent might have introduced some bias caused by the influence on road and urban extent. However, since we rescale the values to be between 1.0·10^−5^ and 1.1·10^−5^ we argue that this effect can be neglected as this is way below the uncertainty in the input data sets. One major additional uncertainty lies in our assumption that the relationship between urban and rural population distribution which we estimate based on data from the start of the century remains valid until the end of the century. Comparable assumptions are also the basis of the input data adding additional uncertainty. These data sets (the input data as well as our data set) should always be seen as our best possible estimate. The user of these data should be aware that the projection will most likely be less accurate for later years compared to earlier years.

## Ethics Statement

The work uses only publicly available data no personal or experimental data was used.

## CRediT authorship contribution statement

**Niklas Boke Olén:** Data curation, Formal analysis, Writing – review & editing. **Veiko Lehsten:** Writing – review & editing, Writing – original draft.

## Declaration of Competing Interest

The authors declare that they have no known competing financial interests or personal relationships which have or could be perceived to have influenced the work reported in this article.
